# Minimising disability in stroke survivors

**DOI:** 10.4103/0972-2327.48871

**Published:** 2009

**Authors:** P. M. Dalal, M. Bhattacharjee

**Affiliations:** Research Centre, C/o Lilavati Hospital, A-791, Bandra Reclamation, Lilavati Hospital & L.K.M.M. Trust Research Centre, A-791, Bandra Reclamation, Mumbai – 400 050, India. E-mail: sanjeev@sctimst.ac.in

Sir,

The Editorial on “Little strokes, big trouble and more” is thought provoking.[1] Stroke prevention by risk factor intervention would be a practical way but we do not have population based national data to plan prevention strategies. To estimate the magnitude of problem, we urgently need population based regional surveys using standard terminologies and methodologies.[2]

On the other hand, Quality of Life in stroke survivors is an immediate problem. For example, in Mumbai Stroke Registry[3] among other factors (e.g. age, stroke subtype and associated risk factors), the neurological deficit (by NIHSS score) at onset correlated with outcome status (by Barthel Index or Modified Rankin Scale) at 28 days. Mild deficit at onset was associated with good recovery whereas; moderate to severe neurological deficit had poor outcome. Therefore, the aim of immediate treatment will be to restrict the extent of brain damage and minimize post-stroke disability.

Unfortunately, lack of public awareness on warning symptoms, transportation difficulties and paucity of acute care beds are major handicaps. With limited number of neurologists in our country, one will have to depend on expertise of local medical practitioners to initiate emergency intensive management (not tPA) until patient is shifted to acute care unit. Thus, training of local physicians in acute stroke care is most urgently needed. In other words, our emphasis on sophisticated advanced technologies requires rethinking and our concern for improving quality of life in stroke survivors needs greater emphasis.
